# Behavioral and neuropathological changes in animal models of chronic painful scar

**DOI:** 10.1007/s00776-013-0453-7

**Published:** 2013-08-21

**Authors:** Yukihiro Kajita, Katsutoshi Suetomi, Teruhiko Okada, Masahiko Ikeuchi, Young-Chang P. Arai, Keiji Sato, Takahiro Ushida

**Affiliations:** 1 Multidisciplinary Pain Center, Aichi Medical University, Karimata, Yazako, Nagakute, Aichi 480-1195 Japan; 2 Department of Orthopedic Surgery, Aichi Medical University, Nagakute, Japan; 3 Department of Orthopedic Surgery, Kochi Medical School, Kochi, Japan

## Abstract

**Background:**

Long-lasting limb pain or back pain after surgery occasionally develops into chronic pain that leads to lower activity and a poorer quality of life for many patients. To determine the histopathological and neuropathological mechanisms that cause persistent post-operative pain, we developed an original animal model with sustained painful scars and then examined pain-related behavior and the pathological alteration of peripheral tissues and spinal nerves associated with the model.

**Methods:**

The animal model (Scar group) was prepared in rats by extensively stripping subcutaneous tissue from the plantar in the hind paw followed by subsequent examination of pain-related behavior over the next 12 weeks. Thereafter, we conducted histological staining of the scar tissues, immunohistochemical staining of c-Fos (L5 dorsal horn), and electron microscopic analysis of the L5 spinal nerve fibers/dorsal roots.

**Results:**

The mechanical pain threshold decreased specifically in the ipsilateral plantar in animals with scar. This state was maintained for 12 weeks. A collagen layer developed from fibers derma to the muscular layer in the scar tissue in which many fibroblasts were observed. No statistical differences were found for the areas of the c-Fos-immunoreactive (c-Fos-IR) neurons in the ipsilateral and contralateral sides of the L5 level of the dorsal horn in both the Scar group and Pinhole (sham operation) group. However, myelin sheath fragmentation of the nerve fibers was observed in the ipsilateral dorsal root at the L5 position.

**Conclusions:**

We created a persistent painful scar model through extensive injury of the peripheral tissues. Fibrotic thickening of the cutaneous tissues, possible sensitization, and partial degradation of the spinal nerve related to the painful scar were observed. This model should enable us to better understand the mechanism of sensitization caused by painful scar and investigate new methods for treating painful scars in humans.

## Introduction

Scar formation is a process that is essential for repairing wounds at the site of trauma and surgical incisions. It has been reported that the scar tissue of patients suffering from failed back syndrome (FBS) is associated with dysfunction of the peripheral nerve [1]. Spinal decompression can generally reduce the pain in patients with sustained low back pain. In some cases, however, the pain still persists after decompression of the nerve, suggesting the possibility that dysfunction of the peripheral nerve at the scar site may be causing the chronic pain. It has also been observed that motor denervation is associated with group muscle atrophy after back surgery [2], which suggests that sensory nerve damage may occur under the same conditions. Another study reported that the chronic pain found after total hip arthroplasty was related to intraoperative damage to the nerve that transversed the surgical field [3].

Several previous animal models of chronic pain based on nerve injuries have contributed to our understanding of the mechanism of neuropathic pain. However, because of a lack of specific animal models, there are currently no useful methods that can be used to understand the underlying mechanisms of chronic pain derived from scar tissue. Recently, we developed a new painful scar animal model that can be used to provoke a reproducible and quantifiable mechanical hyperalgesia that persists during the subacute phase, which lasts for 4 weeks [4]. The purpose of this study was to use this animal model to identify the behavioral characteristics of the pain associated with the scar tissue and investigate the effect of the painful scar formation on the spinal nerve and dorsal horn neurons over a 12-week period. This study used both behavioral and immunohistochemical techniques to investigate changes in the pain-related behavior over time, the pathological alteration of the peripheral tissues and spinal nerve, and the sensitization of the dorsal horn neurons.

## Materials and methods

This investigation was conducted under a protocol approved by the Animal Care Committee of Aichi Medical University.

### Experimental animals and preparation

A total of 26 adult male Sprague–Dawley rats weighing 150 g (Japan SLC) were used in this study. The animals were housed in pairs in plastic cages with soft bedding and allowed free access to food and water. The rats were kept for at least 5 days under these conditions before surgery and were divided into two groups (Pinhole and the Scar groups).

### Surgery

All rats were anesthetized by intraperitoneal injection of pentobarbital sodium (50 mg/kg, Nembutal^®^, Dainippon Sumitomo Pharma).

**Fig. 1:**
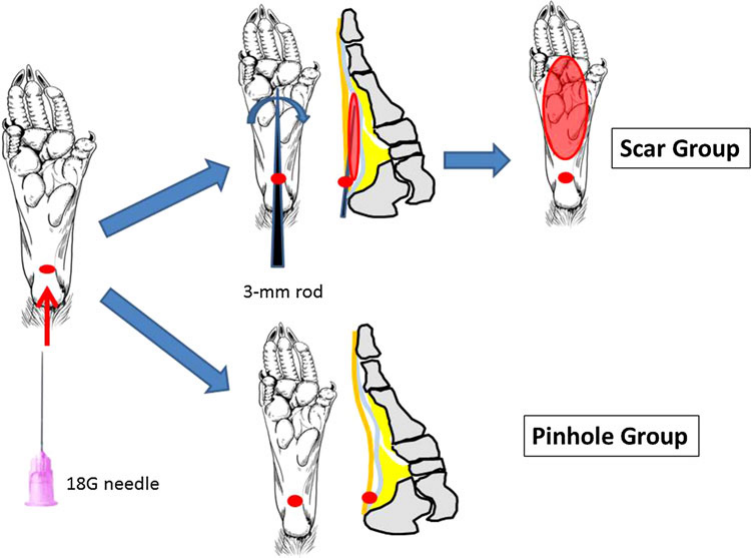
Experimental animals and preparation. The rats were divided into two groups. Scar group: a pinhole was made in the left heel using an 18G needle, with a scar then made by stripping the subcutaneous tissue through the hole using a steel rod (*ϕ* = 3 mm). Pinhole group: a pinhole was made in the left heel using an 18G needle

In the Scar group (painful scar in the foot, *n* = 13), a pinhole was first made in the left heel using an 18G needle. The scar was created by stripping the subcutaneous tissue by use of a steel rod (*ϕ* = 3 mm) that was placed through this hole (Fig. 1). In the Pinhole group (sham operation for the left foot, *n* = 13), a pinhole was made in the left heel using an 18G needle (Fig. 1). No surgical procedures were performed on the right side of any of the animals.

### Behavioral testing

After surgery, the rats were returned to their original cages and maintained under the same conditions used during the pre-operative period. To quantify the mechanical threshold of the hind paw, the occurrence of foot withdrawal in response to graded mechanical stimuli was measured. Mechanical stimuli were applied using von Frey filaments. During the testing, all rats were placed on a metal mesh floor covered by a transparent plastic dome (8 × 8 × 18 cm). Mechanical stimuli were applied to the sensitive area of each hind paw by use of the von Frey filaments. When applied, enough force was used in each case to ensure there was a slight bend in each filament. A single trial of 10 applications with each von Frey filament was carried out within 10 s. A series of trials were then carried out in ascending order starting from the weakest force. A positive response was recorded when a significant withdrawal was observed, with the weakest positive response regarded as the threshold. All of these behavioral tests were carried out before the scar was made (Day 0) and continued every week up until the 12th week after surgery.

### Preparation for histological and immunohistochemical analysis

At 12 weeks after surgery, all rats received an overdose of isoflurane anesthesia (Forane^®^, Abbott) and were transcardially perfused with 4 % paraformaldehyde in 0.1 M phosphate buffer, pH 7.2 (Wako Laboratory Chemicals). The tissue samples, including spinal cords and both feet, were harvested. The removed tissues samples were re-fixed overnight in 4 % paraformaldehyde in 0.1 M phosphate buffer, pH 7.2. To maintain the shape of the samples, the tissue was soaked in increasing concentrations of sucrose (Wako Laboratory Chemicals), starting from 5 to 20 %. Spinal cords and half of the foot samples were rapidly frozen in optical cutting temperature (OCT) compound (Sakura Finetek Japan) to make frozen tissue blocks. All of the sample sections were prepared using a cryostat set at a thickness of 15 μm.

After washing in TBST (tris buffered saline containing 0.1 % Tween 20) three times, sample sections were incubated in blocking solution (4 % Block Ace; DS Pharma Biomedical) for 2 h at room temperature. Sections were then washed three times in washing buffer (0.4 % Block Ace containing 0.1 % Tween 20) followed by incubation with anti-c-Fos antibody (1:1000; Biomol) for 24 h at 4 °C. After washing the sections three more times in the washing buffer, they were subsequently incubated with goat anti-rabbit IgG antibody conjugated with Alexa Fluor 546 (1:400, Invitrogen) at room temperature for 2 h. Finally, all sections were washed five additional times in the washing buffer, and coverslipped after adding ProLong^®^ Gold Antifade Reagent with DAPI (Invitrogen).

All of the six rats in the Scar and Pinhole groups were used for immunohistochemical staining. The spinal cord at the L5 level was sectioned transversely in each animal. After acquiring all the section images, the c-Fos immunoreactive neurons (c-Fos-IR neurons) of the dorsal horn were evaluated. To measure the area of the c-Fos-IR neurons in the ipsilateral or contralateral dorsal horn of the plantars with scars or pinholes, sections from each rat were examined with a deconvolution fluorescence microscope (BZ-9000; Keyence Japan) and analyzed by use of Dynamic Cell Count Image-Analysis software (Keyence Japan). To analyze the location of the c-Fos-IR neurons, the dorsal horn of the spinal cord was divided into two regions: the superficial laminae (laminae I–II), and the deep laminae (laminae III–V).

### Histopathology

The other half of the foot samples were embedded in paraffin (Sakura Finetek Japan), with the sample sections cut at a thickness of 5–10 μm. All the serial sections were stained with hematoxylin–eosin (HE) (Wako Laboratory Chemicals) and Masson trichrome (MT; Sigma–Aldrich) stains for histopathological examination of the scar formed in the left plantar of the hind paw. All five rats in the Scar and Pinhole groups underwent histopathological examinations.

### Electron microscope analysis

Both the L5 spinal nerves and the proximal side of the spinal ganglion were immediately excised and cut into small pieces with a razor blade. Spinal nerves were fixed with 2 % glutaraldehyde and post-fixed with 1 % OsO_4_. Thin sections were stained in uranyl acetate and lead citrate. Sections were examined using a Hitachi (Japan) 7100 electron microscope.

### Statistical analysis

Behavioral results for each of the groups were analyzed by use of the Friedman test with post-hoc analysis. The Wilcoxon matched-pairs test was used to compare the behavioral results for the ipsilateral and contralateral hind paw.

The differences in the area of the c-Fos-IR neurons between the ipsilateral and contralateral sides of the spinal dorsal horn were assessed by use of the Mann–Whitney test. For all analyses, statistically significant differences were expressed as *p* values <0.05.

## Results

### Pain-related behavior

**Fig. 2:**
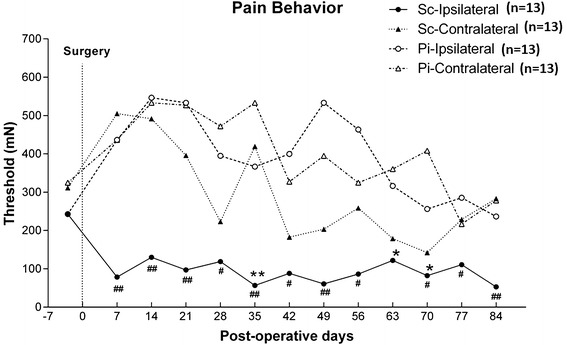
Changes in responses to mechanical stimulation over time. Withdrawal threshold of the hind paw in response to mechanical stimuli was measured by the von Frey test before surgery and every other week after surgery. Data are presented as mean values. *P* values were compared to the preoperation values obtained for each hind paw of both groups at each of the time points shown (*P < 0.05, **P < 0.01). Withdrawal threshold of the ipsilateral hind paw decreased at 5, 9, and 10 weeks after surgery in the Scar group. Withdrawal threshold of the treated hind paw was compared with the contralateral side at the same time point in both the Scar and Pinhole groups. The withdrawal threshold on the ipsilateral (treated) side was significantly lower at 1, 2, 3, 4, 5, 6, 7, 8, 10, 11, and 12 weeks after surgery in the Scar group (^#^ *P* < 0.05, ^##^ *P* < 0.01) compared with the contralateral side of the same animal. (*Sc* Scar group, *Pi* Pinhole group)

Changes in the withdrawal threshold over time to the mechanical stimulation by use of the von Frey filaments are shown in Fig. 2. Compared with the pre-operative control value, no significant threshold changes were observed at any of the time points after surgery for either the hind paws of the Pinhole group or the contralateral side of the hind paws of the Scar group. However, withdrawal threshold of the ipsilateral hind paw decreased at 5, 9, and 10 weeks after the surgery in the Scar group. In addition, compared to the contralateral side of same animal, the withdrawal threshold on the ipsilateral (treated) side was significantly lower 1, 2, 3, 4, 5, 6, 7, 8, 10, 11, and 12 weeks after surgery. Comparisons between the Pinhole and Scar groups indicated that there was a decreased withdrawal threshold for both the ipsilateral hind paws and the contralateral hind paws at all of the time points after the surgery.

### Histopathological examination of the scarred plantar

**Fig. 3:**
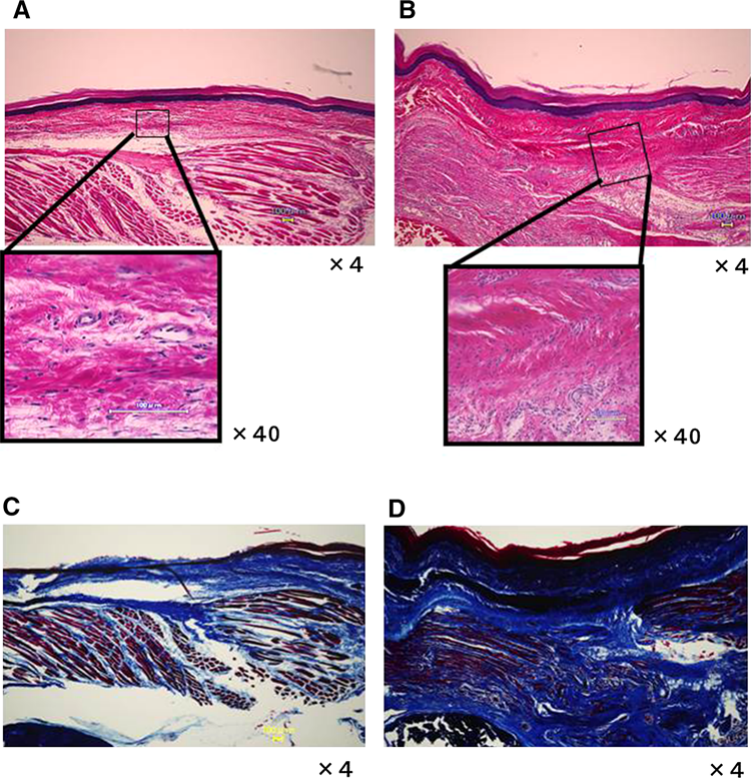
Histopathological analysis of the foot scar tissue. Plantar tissues were harvested at the 12th week after surgery, with the paraffin-embedded sections stained with either hematoxylin–eosin (HE, *upper panel*) or Masson trichrome (MT, *lower panel*) stains. Ipsilateral plantar tissues of the Pinhole (**a**, **c**) and the Scar (**b**, **d**) groups are shown. The dermis was thicker compared with the control side, and contained a larger number of collagen fibers. The boundary between the dermis and the subcutaneous area was not clear. Additionally, there are migrating cells that have round or oval nuclei between the collagen fibers in the Scar group (**b**). The collagen fibers run in random directions and there are also many collagen fibers present in the dermis and subcutaneous layer in the Scar group (**d**)

Compared with the Pinhole group, the dermis and epidermis in the Scar group became thicker and contained a larger number of collagen fibers. The sizes of collagen bundles varied, with the boundary between the dermis and subcutaneous area found to be unclear. Many migrating cells with round or oval-shaped nuclei were observed between the collagen fibers (HE staining, Fig. 3a, b). In contrast with the Pinhole group, the Scar group exhibited an aggregation of collagen fibers in the dermis and subcutaneous layer. These collagen fibers ran in random directions (MT staining, Fig. 3c, d). Similar histological findings were observed in each of the five rats of the Scar and Pinhole groups.

### Immunohistological examination of the spinal dorsal horn

**Fig. 4:**
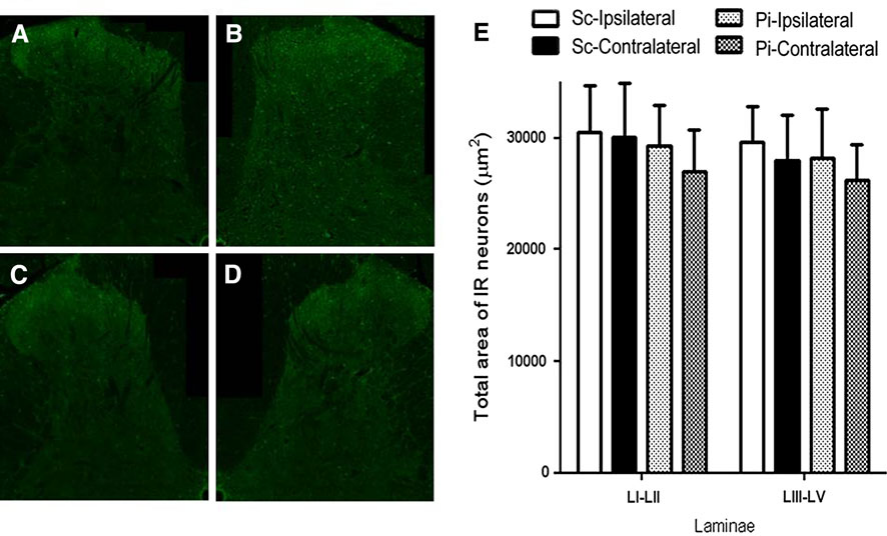
Representative expression of c-Fos-IR fibers in the dorsal horn of the spinal cord. **a** The contralateral side of the dorsal horn in the Scar group. **b** The ipsilateral side of the dorsal horn in the Scar group. **c** The contralateral side of the dorsal horn in the Pinhole group. **d** The ipsilateral side of the dorsal horn in the Pinhole group. **e** Histogram of the c-Fos-IR fiber count in the spinal dorsal horn 12 weeks after the operation. (*Sc* Scar group, *Pi* Pinhole group, *L I–II* Laminae I–II, *L III–V* Laminae III–V) Compared with the spinal dorsal horn on the contralateral side of the Scar group and the spinal dorsal horn of the Pinhole group, there were no statistical differences between the Scar and Pinhole groups for the areas of the c-Fos-IR neurons in the ipsilateral and contralateral sides of the Lamina I–II and III–V of the dorsal horn

There was no statistical difference between the Scar and Pinhole groups for the areas of the c-Fos-IR neurons in the ipsilateral and contralateral side of the L5 level Lamina I–II of the dorsal horn. Similarly, there was no statistical difference between these groups for the areas of the c-Fos-IR neurons in ipsilateral and contralateral side of the L5 level Lamina III–V of the dorsal horn (Fig. 4).

### Electron microscopy analysis of nerve fibers

**Fig. 5:**
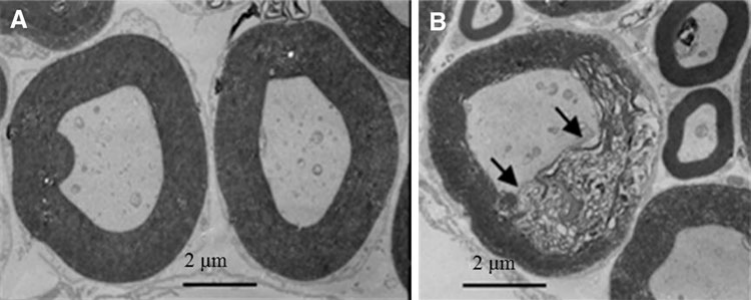
Electron microscopy analysis of the nerve fibers. Nerve fibers in the spinal dorsal horn at L5 were analyzed by transmission electron microscopy at 12 weeks post-surgery (**a** Pinhole group, **b** Scar group). Nerve fibers in the Pinhole group were of normal shape. The myelin sheath of the nerve fibers in the Scar group was fragmented (*indicated by the arrow*), and the Schwann sheath and axon were intact

Nerve fibers in the control side appeared normal. No degeneration of the axon and the myelin sheath was observed. The plasma membrane of the axon was in intimate contact with the myelin sheath (Fig. 5a). In contrast, nerve fibers in the scarred side showed myelin sheath fragmentation, while the Schwann sheath and axons were intact. The plasma membrane of the axon was not in intimate contact with the myelin sheath (Fig. 5b).

## Discussion

A scar in the skin is defined as “a macroscopic disturbance of normal structure and function of the skin architecture, resulting from the end product of a healed wound” [5]. Therefore, scarring is an abnormal skin structure in which there is a failure to regenerate as opposed to a biomechanical problem [6]. Additionally, a scar is believed to be one of the factors associated with chronic pain. In a previous animal study, Henderson et al. [7] suggested that increased densities of substance-P and calcitonin gene-related peptide (CGRP) in healing wounds may have a role in the unpleasant sensory symptoms associated with these healing wounds. Sulaiman et al. [8] identified substance-P and CGRP in peritoneal adhesions of patients who suffered from chronic pain after laparotomy. Although we did not investigate the peripheral/local neuropeptide reactivity in this report, we have determined there was up-regulation of substance-P and CGRP in the ipsilateral spinal dorsal horn in a previous study [4]. Thus, similar neuropathological changes might have existed in this study, as the histopathological findings for the foot on the scar side showed hypertrophic scars that were presumed to be painful.

Several types of chronic pain animal models have been developed and used to study the development and maintenance of chronic pain mechanisms. Interestingly, several chronic pain models, such as the neuropathic pain model [9], muscle pain model [10], joint pain model [11], and the joint immobilization model [12], have all shown that there was bilateral hyperalgesia in previous behavioral and immunohistochemical studies of the spinal cord. In this study, the withdrawal threshold of the ipsilateral hind paw was lower than the pre-surgery value, although this change was not observed on the contralateral side. Since there was a decrease in the withdrawal threshold for the contralateral side of the Scar group compared to the Pinhole group (both ipsilateral and contralateral side) at the same time points, this suggests that changes in sensory functions may occur on both sides in our Scar model animal.

Recently, c-Fos expression was used as a functional marker for identifying activity in spinal neurons in response to noxious stimulations [13, 14]. Moreover, not only noxious stimulation, which includes thermal, mechanical and chemical stimuli, but also non-noxious stimulation can be used to provoke the expression of c-Fos in the brain and spinal cord. Therefore, cautious interpretations are required when drawing conclusions from the results of studies examining c-Fos expression [13]. Since decreased withdrawal thresholds were observed at almost all of the time points examined in our present behavioral study, we predicted that there was upregulation of c-Fos expression in the ipsilateral (treated) side of the dorsal horn. However, we found no statistical difference between the Scar and Pinhole groups for the areas of the c-Fos-IR neurons in the ipsilateral and contralateral sides of the L5 level of the dorsal horn. Overall, our results suggest that the chronic pain behavior mechanisms in our model are not simple and that the c-Fos positive sensitized spinal dorsal horn neurons are not crucial for this pain mechanism. Therefore, it is essential that further research be undertaken so that the underlying mechanisms that provoke behavioral changes in this chronic scar model can be clarified.

Our study also demonstrated that the spinal nerve fibers in the scarred side were an indication of fragmentation of the myelin sheath. Moreover, this model showed there was ipsilateral mechanical hyperalgesia occurring at same time. Fragmentation of the myelin sheath is one of the histological findings of Wallerian degeneration. When peripheral nerves are injured, macrophages are known to be recruited from the blood to the damaged nerve, where they then make a significant contribution in the removal of degenerating axons and myelin sheaths and in the process of regeneration [15]. While the peripheral nerve normally contains a small population of resident macrophages, consisting of 1–4 % of the cell population in the rat sciatic nerve [16], this small population of resident macrophages is not sufficient for causing degradation of the myelin sheaths following nerve injury. It has also been suggested that Wallerian degeneration is linked to the pathogenesis of neuropathic hyperalgesia [17]. Although peripheral nerves were not directly injured in our study, the process of Wallerian degeneration might have existed in the spinal nerve and, thus, these changes could have been linked to the sustained scar pain behavior that was observed in this experiment.

In conclusion, we developed an animal model of painful scar that develops hyperalgesia at the 12th week following surgery. These effects might be the result of plastic changes in the central nervous system that were caused by sustained pain. Our model should enable better understanding of the mechanism of sensitization caused by painful scar and, in the future, help us to investigate new methods for treating painful scar in humans.
